# Drug-induced gingival overgrowth: evaluation of an online survey

**DOI:** 10.1007/s00210-026-05600-6

**Published:** 2026-06-20

**Authors:** Philipp Friedrich Georg Ried, Roland Seifert

**Affiliations:** Institute of Pharmacology, Hannover Medical School, 30625 Hannover, Germany

**Keywords:** Gingival disease, Gingival overgrowth, Drug-induced gingival overgrowth, Drug-related side effects and adverse reactions, Surveys and questionnaires, Dentists, Germany

## Abstract

Drug-induced gingival overgrowth (DIGO) is becoming an increasingly important issue due to high prescription rates of inducing pharmaceuticals. Therefore, the main focus of this study was to determine what experiences dentists in Germany have had with DIGO and which active ingredients cause this ADR in their patients. The conducted online survey was sent to all 17 German dental associations asking them to forward the survey to their members. Five associations complied with the request. Out of 25,562 dentists who were potentially eligible, 118 questionnaires were evaluated, resulting in a response rate of 0.46%. Furthermore, the 24 questions were designed to shed light on the frequency with which this adverse drug reaction (ADR) occurs, as well as patient factors, treatment strategies and potential risks. Amlodipine, nifedipine and ciclosporin were cited as the most frequent triggers of DIGO. In addition, no gender-specific differences were found among the affected patients. Sixty-nine percent of respondents were able to successfully treat this condition, using the following treatment options in descending order: “oral hygiene instruction”, “change in medication”, and “surgical procedures”. Accordingly, it is difficult to determine a precise prevalence with which a particular active substance leads to gingival overgrowth. It is therefore advisable to specify a narrow percentage range with which the prevalence can be reliably described. In addition, the underlying parameters of the source under consideration should be examined very carefully, as different sources may come to very different results.

**Supplementary Information:**

The online version contains supplementary material available at https://doi.org/10.1007/s00210-026-05600-6.

## Introduction

Gingival overgrowth (GO), previously referred to as gingival hyperplasia or gingival hypertrophy, represents a clinically relevant adverse drug reaction (ADR) associated with various pharmacological agents. Anticonvulsants, calcium channel blockers, and immunosuppressants are considered the drug classes most frequently implicated in the development of this condition (Brown and Arany 2015). Beyond pharmacotherapy, additional etiological factors such as systemic diseases, genetic predisposition, allergic reactions, local irritants, hormonal fluctuations, and vitamin deficiencies have been identified as potential contributors to gingival overgrowth (Beaumont et al. 2017; Moffitt and Cohen 2013; Seymour et al. 2000). The precise molecular mechanisms underlying drug-induced gingival overgrowth (DIGO) have not yet been fully clarified. A commonly proposed pathophysiological pathway describes drug-induced inhibition of sodium and calcium channels, resulting in reduced intracellular ion influx. This alteration is assumed to impair folic acid uptake, leading to reduced activation of collagenases. Consequently, collagen degradation is reduced, disrupting the physiological balance between extracellular matrix synthesis and degradation and promoting connective tissue accumulation (Droździk and Droździk 2023). In addition to drug-specific mechanisms, it is assumed that various cytokines play a significant role in the development of DIGO (Trackman and Kantarci 2004). Compared to other areas of the body, interdental papillae are particularly susceptible to overgrowth-inducing drugs due to their specific surface structure and composition (Ramírez-Rámiz et al. 2017).

Clinically, pronounced gingival overgrowth is associated with aesthetic impairment, compromised oral hygiene, exacerbation of inflammatory processes, pain, bleeding, and masticatory dysfunction (Bharti and Bansal 2013). Progressive gingival inflammation may contribute to the development of periodontitis, which in turn can promote systemic inflammatory responses and ultimately result in tooth loss (Chojnacka-Purpurowicz et al. 2022).

Possible therapeutic approaches are improvement of dental and oral hygiene including subgingival instrumentation, as well as regular professional dental cleaning. Another approach is the change or discontinuation of the causative medication, if possible and appropriate, and surgical removal of excessive tissue to create hygienic conditions (Tungare and Paranjpe 2024). Unfortunately, drug-induced gingival overgrowth (DIGO) has a high recurrence rate, requiring regular surgical tissue removal every 12–18 months (Zoheir and Hughes 2019). Other treatment options discussed in recent publications include the topical application of folic acid and the systemic use of antibacterial agents such as azithromycin (Arya et al. 2011; Fuchs et al. 2019). The best-known drugs to induce DIGO are phenytoin, nifedipine, ciclosporin and amlodipine (Brown and Arany 2015). However, diltiazem, verapamil, valproic acid and carbamazepine also trigger this adverse reaction (Miranda et al. 2005).

In addition, reliable prevalence rates are difficult to find, as the information from different sources on one and the same substance varies greatly (Droździk and Droździk 2023).

Due to the circumstances described above, this study aims to provide a comprehensive picture of which drugs are most likely to cause gingival overgrowth and what possible consensus currently exists among German dentists.

To obtain a comprehensive picture of the current situation in dental offices in Germany, an online survey was conducted from February to May 2024. Among other findings, the survey revealed that dentists regularly encounter patients suffering from gingival overgrowth. Therefore, it is suspected that more patients currently suffer from gingival overgrowth and DIGO in particular than the low prevalence of this ADR and information from PIs and SmPCs would suggest. Similar results are also shown in the study by Cyris et al. (2024), who also conducted an online survey among dentists in Germany. Cyris et al. (2024) likewise emphasise an interdisciplinary approach to patient-specific therapy with the aim of switching or discontinuing, triggering agents prescribed by physicians or other specialised practitioners in collaboration with the treating dentist, if possible.

## Materials and methods

### Online survey

An online survey was conducted among German dentists to record their daily experiences with drug-associated gingival overgrowth, its cause, frequency and treatment. Both single and multiple-choice questions were asked. During the development of the questionnaire, questions were formulated that were as easy to understand as possible in order to shed light on various topics. The focus was upon identifying drugs that dentists increasingly associate with gingival overgrowth in their daily practice and determining treatment strategies that lead to successful outcomes. In addition, information was collected on possible risk groups, observed symptoms, current medication and mechanisms of action. The authors’ aim was to use a total of 24 questions to paint a comprehensive picture of the current situation of German dentists in dealing with DIGO.

To conduct the survey, all 17 German dental associations were asked to distribute the questionnaire to their members via email. A total of five associations agreed to the project and distributed the questionnaire along with a short cover letter explaining the project and describing how the results would be used in this paper. The dental associations of the following federal states sent the questionnaire to their members by email:

The survey was sent to all dentists registered at the respective dental associations. In order to achieve the largest possible participant base, no further exclusion criteria were specified. Data from the German dental association from 2023 provides an overview of the number of members practising dentistry and those currently not practising dentistry per association:

Assuming that the survey was sent to all members of the respective dental associations by email, both practising dentistry and currently not practising dentistry, the total number of potential participants is 25,562. However, it is unclear whether all potential members were actually reached by email and whether they checked their emails during the survey period. Calculating the non-response rate from 25,562 participants and 118 returned questionnaires, this equals 99.53%. Therefore, the corresponding response rate is 0.4616%.

The online survey was completed anonymously in its entirety via “SoSci Survey”. As the survey by design did not aim for the collection of personal data and could not collect such data, there were no data protection requirements to be observed. The questionnaire is attached in the supplement.

**Fig. 1:**
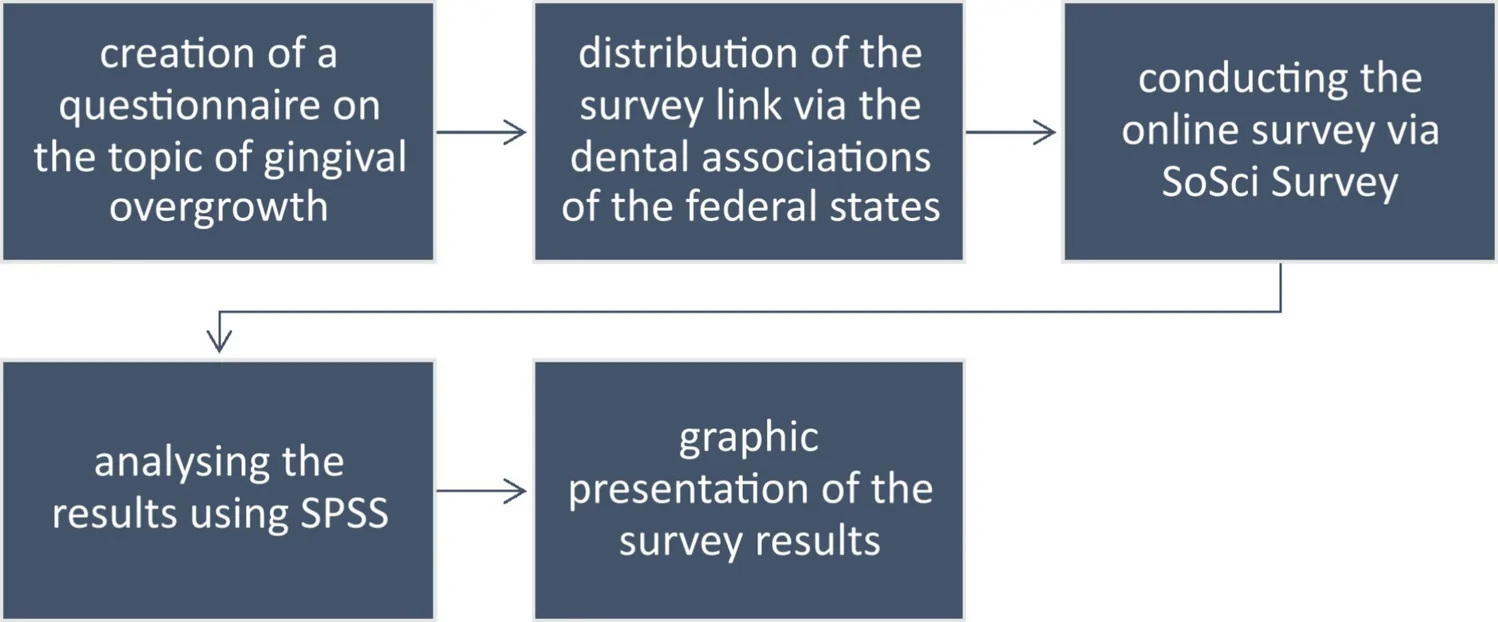
Presentation of the underlying methods used for this paper on the topic of gingival overgrowth

In the survey period from 19 February 2024 to 2 May 2024, 84 fully completed and 34 incomplete questionnaires were recorded. A total of 118 questionnaires were statistically analysed using SPSS, and a descriptive analysis of the data collected was carried out (Fig. 1).

## Results

The questions asked in the survey and the responses collected are presented and analysed below.

‘Do you suspect that the gingival overgrowth could potentially be drug-induced/associated?’

Eighty-four percent of respondents stated that they suspected that the gingival overgrowth experienced by their patients could be drug-induced. Only 6% denied a drug association, while 10% answered the question with ‘unknown’. The results of this question illustrate how often a drug is suspected as the cause of gingival overgrowth (Fig. S1). This indicates that only a small percentage of the proliferations are caused by the abovementioned non-drug triggers.

‘Were you able to determine the cause of the gingival overgrowth?’

Looking at the trigger of the proliferation, this could be determined in 63.2% of cases; in 17.9%, this was not possible; and in 18.9%, the trigger remains unknown (Fig. S2). In order to reduce the number of unknown and undetectable cases, additional training and information programmes would certainly be helpful.

‘Which drug in particular was taken by the patient at the time of the onset of gingival overgrowth? (multiple choice possible)’

**Fig. 2:**
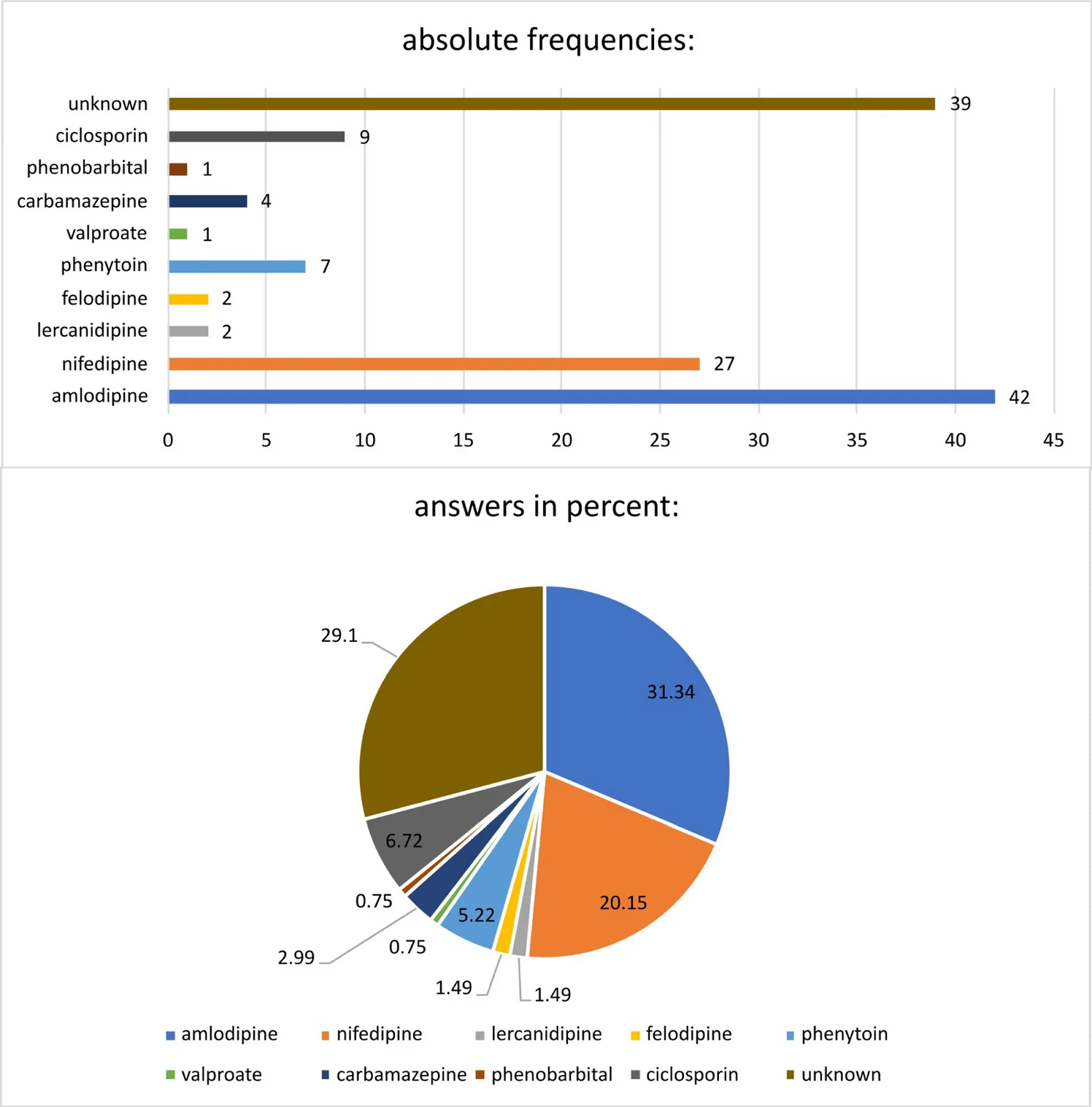
Evaluation of the specific active substance taken. Presentation of registered responses in per cent and absolute numbers

In terms of the specific drugs taken by patients, amlodipine leads the way with 31.34%, followed by nifedipine with 20.15%. This is followed by ciclosporin with 6.72%, phenytoin with 5.22%, carbamazepine with 2.99%, felodipine and lercanidipine with 1.49% each and finally phenobarbital and valproate with 0.75%. In 29.1% of cases, the drug taken was unknown (Fig. 2). These results could also be explained by both the frequency of prescription and the prevalence with which they lead to gingival overgrowth.

‘Are there specific patient groups in which you observe an increased incidence of gingival overgrowth?’

Of course, it is also relevant whether there are certain patient groups in which gingival overgrowth can be observed more frequently than in others. In fact, 60.2% of respondents said yes to an increased occurrence of this adverse drug reaction in certain patients. Twenty-three percent of respondents negated the question and 16.8% stated ‘unknown’ (Fig. S3).

‘In your experience, are women or men more frequently affected by gingival overgrowth?’

No gender-specific difference was found in the distribution of patients. Thirty-eight percent of respondents stated that ‘men and women are affected about equally often’. 21.2% considered that ‘men are affected more often’, while 20.4% thought that ‘women are affected more often’. Another 20.4% of respondents answered ‘unknown’ (Fig. S4).

‘In your opinion, which of the following factors is the most important risk factor for the development of gingival overgrowth?’

**Fig. 3:**
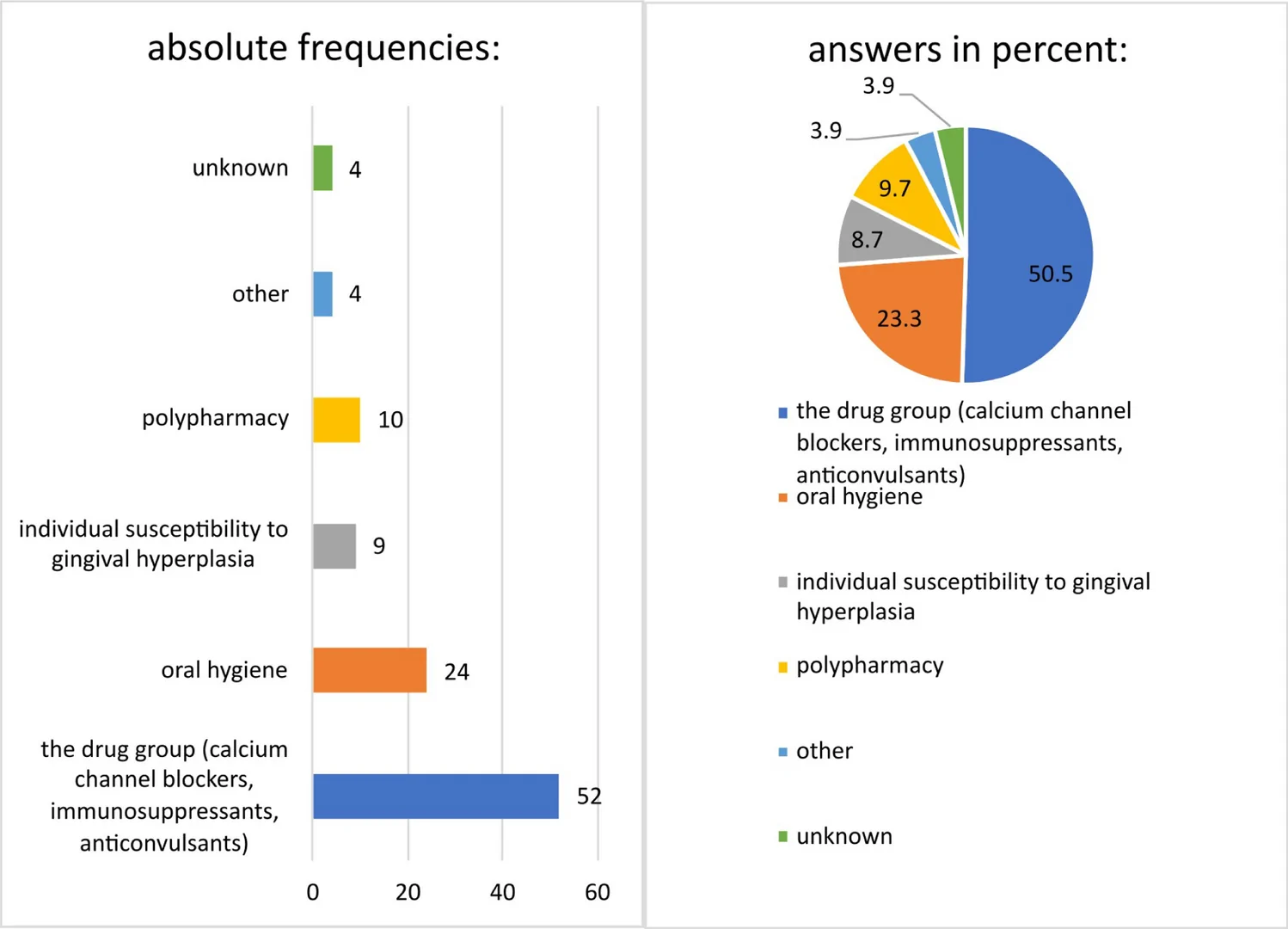
Overview of the most important risk factor for the development of gingival overgrowth according to the respondents. Presentation of the answers in per cent and absolute numbers

At 50.5%, the most important risk factor leading to the development of gingival overgrowth is the ‘medication group’ taken by the patient. This is followed by individual ‘oral hygiene’ with 23.3% and ‘polypharmacy’ with 9.7%. In descending order, ‘individual susceptibility’ with 8.7%, ‘other’ with 3.9% and ‘unknown’ factors were also mentioned with 3.9% (Fig. 3).

‘Was your therapy successful?’

Encouragingly, 69.3% of respondents stated that their treatment was successful. 12.9% did not treat the overgrowth themselves and in only 8.9% of cases was the treatment unsuccessful or the outcome unknown (Fig. S5).

‘Which treatment options do you use for your patients with gingival overgrowth? (multiple choice possible)’

This raises the question of which therapy the patients were treated with. In 36.7% of cases, ‘oral hygiene instructions’ were given, followed by a ‘change of medication’ in 25.5%. In 22.8%, the growth was treated ‘surgically’ and in 10.3% ‘wait and see’. In 4.4%, a ‘different’ treatment option was chosen (Fig. S6). According to current scientific findings, this corresponds to the recommended treatment regime. Starting with an improvement in oral hygiene, professional tooth cleaning should be carried out, followed by a change in medication if possible. In particularly pronounced cases, as well as in recurrences, the proliferating gingiva should be surgically removed (Tungare and Paranjpe 2024).

‘Has the gingival overgrowth subsided or completely regressed after stopping/changing the medication?’

**Fig. 4:**
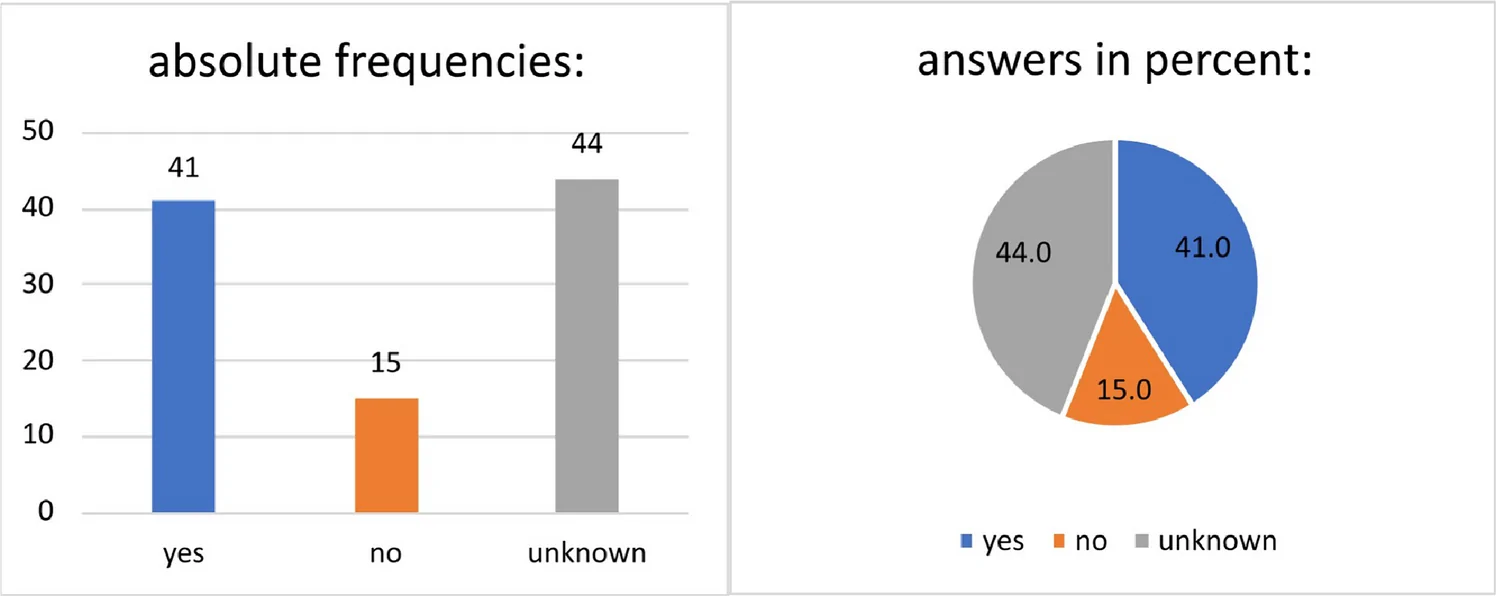
Overview of the answers given to the question as to whether the gingival overgrowth subsided when the medication taken was discontinued/changed. Presentation of the results in per cent and absolute numbers

In 41% of cases, an improvement and/or a complete reduction in gingival overgrowth was observed after switching medication. In 15%, there was no improvement, and in 44%, the effect was unknown. This illustrates the effectiveness of a drug switch as a treatment option if possible (Fig. 4).

‘Which drug class does the triggering drug that led to the occurrence of gingival overgrowth in your patients belong to?’

If we look at the drug classes available for selection, which were being taken at the time of the proliferation, we see that 53.9% stated calcium channel blockers. Anticonvulsants and combinations of several drug classes were named by a clear gap at 9% each. Immunosuppressants were mentioned by 7.9% and other drug classes by 2.2%. In 18% of cases, the triggering drug class was unknown (Fig. S7). The reason for the significantly more frequent mention of calcium channel blockers could be, on the one hand, the very high prescription rate of these drugs and, on the other hand, possibly a high prevalence of gingival overgrowth triggered by this class of drugs. It is probably a combination of both factors.

‘In your experience, which drug most frequently triggers gingival overgrowth?’

Irrespective of the previous question, 30.3% of respondents stated that amlodipine is the most common trigger for gingival overgrowth. 11.2% stated that nifedipine was the most frequent trigger; 5.6% named ciclosporin. 4.5% of respondents named phenytoin as the most frequent trigger and 1.1% phenobarbital. Again, calcium channel blockers are among the most common triggers of gingival overgrowth, followed by immunosuppressants and anticonvulsants (Fig. S8).

‘How often have patients with gingival overgrowth already consulted you?’

To illustrate the relevance of drug-associated gingival overgrowth, respondents were asked about the frequency with which patients presented themselves to the dentist regarding this problem. At 46%, ‘occasionally’ was the most frequently chosen answer, followed by ‘rarely’ at 29.2%. ‘Frequently’ was given by 20.4%, ‘very often’ by 2.7% and ‘never’ by 1.8%. These results show that if ‘occasionally’ and ‘often’ are taken together, a large proportion of dentists are regularly confronted with drug-associated gingival overgrowth (Fig. S9). Accordingly, it is more important to be aware of possible triggers and the appropriate therapy in order to be able to help patients in the best possible way.

‘Which age group do your patients with gingival overgrowth belong to?’

In order to further narrow down possible risk groups, the above shown question was asked about patient age. The largest group of patients observed, at 23%, was between 40 and 59 years old and 65 years and older. Subsequently, patients between 60 and 64 years of age were specified with 16.8%, followed by 26–39 year-olds with 9.7%. Patients between 18 and 25 years were described with 8% and the smallest group with 4.4% was between 6 and 17 years old. This age distribution clearly shows the correlation between increased intake of various medications with increasing age and the occurrence of gingival overgrowth (Fig. S10). In addition, other factors play a relevant role with increasing age in relation to proliferation, for example, a decline in oral hygiene, an increase in general illnesses and hormonal changes in the body.

‘In your experience, which symptoms frequently occur in patients with drug-induced gingival overgrowth? (multiple choice possible)’

A wide range of symptoms were described by patients with drug-associated gingival overgrowth. In addition to ‘swollen gums’ (23.4%), ‘bleeding gums’ (19%), ‘impaired oral hygiene’ (14.7%), ‘reduced aesthetics’ (12.6%) and ‘painful gums’ (11.8%) were particularly common. Patients also suffered from ‘bad breath’ in 8.1%, “discomfort” in 4.4%, ‘intraoral burning’ in 2.9% and ‘difficulty eating’ in 1.3% of cases. There were ‘other symptoms’ in 0.7% and none of the listed symptoms in 0.5%. The sheer number of symptoms reported and their varying severity illustrate the not insignificant reduction in quality of life caused by gingival overgrowth (Fig. S11).

‘Which of the following mechanisms would you select as the most likely for the development of gingival overgrowth?’

Looking at possible underlying mechanisms, it is striking that 57.1% of people surveyed were not aware of any mechanism that could lead to gingival overgrowth. Nineteen percent stated that it is a ‘combination of the mechanisms mentioned’. 7.1% see an ‘inflammation-induced upregulation of cytokines’ as the trigger and 6% ‘plaque-associated inflammation of the soft tissue.’ In each case, 3.6% selected ‘impaired connective tissue breakdown’ and ‘triggering of inflammatory processes by toxic drug levels’ as the cause. 2.4% of respondents suspected ‘another mechanism’ as the trigger and 1.2% ‘a reduced cation current due to the medication’ (Fig. S12). Even though the underlying mechanism has still not been conclusively researched at the molecular level, there is a general consensus on the following causal cascade: A drug-induced reduced cation influx leads to an intracellular folic acid deficiency, which results in a reduced activation of collagenases. This results in reduced connective tissue degradation and an imbalance between the formation and degradation of extracellular matrix (Droździk and Droździk 2023).

Comparing the different results from the information in the package leaflets with the results of the survey of dentists and current scientific literature (Droździk and Droździk 2023), large differences can be revealed (Table 1). For example, the model calculation using the data from the package leaflet for valproic acid shows that 81.09% of patients receiving long-term valproic acid therapy in Germany would develop DIGO per year, while only 0.75% of the dentists surveyed had patients with gingival overgrowth taking valproic acid. The median of the results of the study analysed is 30.75% for valproic acid. This huge contrast suggests that the survey results must be far from reality. The reason for this extremely low result cannot be conclusively clarified. Lack of knowledge, low local prescription rates or patient-specific factors may be the contributing factors.

**Table 1:** Presentation of the different results for the corresponding active substances. The following abbreviations are used in Table 1: *DIGO* drug-induced gingival overgrowth, *SmPC* summary of product characteristics, *NR* not reported. The results of the package leaflets/SmPC show the percentual distribution of the total expected patients with DIGO per year divided into the corresponding active substances. This was based on the AVR from 2023 and the prevalences stated in the manufacturers’ SmPCs 1, 2 and 3. The results of the survey show the percentual distribution of responses to the question of which drug was taken by patients with gingival overgrowth. The results of the literature refer to the work of Droździk and Droździk (2023)

**Drugs**	**Manufacturer**	**Distribution of expected DIGO cases per year according to package insert/SmPC**	**Distribution of all registered survey answers of which drug leads to DIGO**	**Median prevalences of DIGO for each drug according to ** Droździk and Droździk (2023)
Valproic acid	1	81.09%	0.75%	30.75%
Ciclosporin	1	10.75%	6.72%	33.65%
Amlodipine	1	6.99%	31.34%	48.25%
Phenytoin	2	0.62%	5.22%	42.70%
Nifedipine	3	0.27%	20.15%	44.65%
Verapamil	1	0.14%	NR	19%
Felodipine	1	0.12%	1.49%	NR
Carbamazepine	NR	NR	2.99%	23.55%
Diltiazem	3	0.02%	NR	11.10%

However, if we look at ciclosporin, the results from the package leaflet and the survey are much closer at 10.75% and 6.72%, respectively. The literature provides the highest value here at 33.65%. In the case of amlodipine, the data from the package insert indicates that 6.99% of patients receiving long-term therapy would suffer from gingival overgrowth per year, while 31.34% of the dentists surveyed observed patients with DIGO while taking amlodipine. The study used for comparison rather confirms the survey data at 48.25%. Looking at phenytoin, the data collected are also far apart. According to the package leaflet considered, 0.62% of patients receiving long-term treatment with phenytoin should develop DIGO per year, while the survey shows 5.22% and Droździk and Droździk assume 42.7%. The situation is similar with nifedipine. The result of the package leaflet is again the lowest with 0.27%, the survey is 20.15% and the analysed study shows 44.65%. For verapamil, only the results of the package leaflet, 0.14%, and the study, 19%, could be compared with each other. Felodipine is one of the active substances with the lowest expected frequency of triggering gingival overgrowth, at 0.12% from the package leaflet data and 1.49% from the survey data. For carbamazepine, the data from the package leaflet indicate that 0.08% of patients receiving long-term therapy should develop DIGO per year, while the survey showed 2.99% and the scientific literature 23.55%. For diltiazem, the result from the data in the package leaflet is 0.02% and the study gives a median of 11.1%. It can therefore be seen that, depending on the underlying source, either valproic acid or amlodipine is the active ingredient with the highest prevalence of triggering gingival overgrowth. The coding of the analysed manufacturers in Table 1 can be found in the supplement (Table S1).

To further analyse the results presented above, Table 2 shows the current prescription figures taken from the AVR 2024 in relation to the prevalences recorded in the survey. Given the current amount of amlodipine prescribed, it does not seem surprising that these patients in particular often present themselves to their dentist with gingival overgrowth. If we take the prevalences from the survey as a basis, we arrive at over 2 million patients per year who should suffer from amlodipine-induced gingival overgrowth. In contrast, with lercanidipine, the second most frequently prescribed drug from the list analysed, only 29,404 patients are expected to develop DIGO under long-term therapy. Nifedipine, however, shows an astonishingly high prevalence from the survey of dentists, which results in a correspondingly high number of expected DIGO cases. Overall, according to the current prescription figures, special attention should be paid to the frequently prescribed drugs in order to be able to recognise an adverse drug reaction such as gingival overgrowth triggered by these active substances in sufficient time (Table 2). Nevertheless, it must be mentioned that deriving reliable prevalences out of a sample size of 118 questionnaires is almost impossible. Instead, the data presented should not be interpreted as absolute prevalence rates, but rather as an indication of the frequency with which the dentists surveyed recognised the various active ingredients as triggers. Therefore, it appears that various active ingredients such as ciclosporin, amlodipine and nifedipine were very frequently identified by respondents as triggers for DIGO, while others such as valproic acid or carbamazepine were identified only very rarely. This could be due to the fact that the first-mentioned active ingredients are probably much better known to dentists in connection with DIGO than the later ones. On the other hand, local differences in prescription frequencies may also play a significant role.

**Table 2:** Presentation of the current drug prescription figures in Germany from the AVR 2024. The following abbreviations are used in Table 2: *DDD* defined daily dose, *DIGO* drug-induced gingival overgrowth. Calculation of patients with long-term therapy in Germany using the annual DDDs divided by 365, as well as calculation of the expected DIGO cases per year in Germany using numbers of patients receiving long-term therapy and using the distribution of survey answers as the recognition frequency among respondents. The expected number of DIGO cases shown in the table is provided solely to illustrate the order of magnitude and does not correspond exactly to the actual number of cases

Drugs analysed	Recent DDDs in Germany	Number of patients with long-term therapy in Germany	Distribution of survey answers	Illustrative DIGO cases (model calculation)
Amlodipine	2,387,200,000	6,540,273	31.34%	2,049,721
Nifedipine	12,900,000	35,342	20.15%	7121
Ciclosporin	6,600,000	18,082	6.72%	1215
Phenytoin	3,400,000	9315	5.22%	486
Carbamazepine	26,800,000	73,424	2.99%	2195
Felodipine	36,900,000	101,095	1.49%	1506
Lercanidipine	720,300,000	1,973,424	1.49%	29,404
Phenobarbital	2,300,000	6301	0.75%	47
Valproic acid	48,400,000	132,602	0.75%	994

## Discussion

Different approaches were chosen in the surveys considered in Table S2. In the studies by Moride et al. (1997) and Alvarez-Requejo et al. (1998), the main focus was the comparison between publicly reported adverse drug reactions in pharmacovigilance centres and ADRs identified via the survey. All ADRs observed in a defined time period were registered and compared with the data from the pharmacovigilance centres. Both studies found that significantly more ADRs were reported via the survey than to the pharmacovigilance centres. Under-reporting was lowest for severe, unlabelled ADRs of recently marketed drugs. In contrast, our present study analysed a single ADR, gingival overgrowth. The survey asked specifically about triggering agents and associated influencing factors. The data collected was used to identify the active substances that have a particularly high risk of triggering gingival overgrowth. In addition, the prevalences that emerged from the survey were compared with package inserts, SmPCs and scientific literature. All studies show a higher result of observed adverse drug reactions than presented in public pharmacovigilance centres (Table S2). This can primarily be attributed to under-reporting and should definitely be taken into account when presenting the prevalence of ADRs in postmarketing surveillance.

Online surveys make it possible to compare the findings from current studies and the often very theoretical assumptions with experiences from the day-to-day work of a specific group of people or professions. In this way, theory and practice can be brought together and reliable results can be achieved. In addition, surveys enable the involvement of experts who are confronted with the problems under investigation on a daily basis and who would not have been taken into account by the scientific community without this survey (Evans and Mathur 2005).

The survey conducted depicts that drug-induced gingival overgrowth in particular is a highly relevant topic among German dentists today (Fig. S1, S9). This condition is often accompanied by a variety of symptoms that determine the level of suffering experienced by patients (Fig. S11). Accordingly, the search for the cause, the correct treatment and the successful resolution of this problem is an important part of dental work.

The clinical diagnosis of DIGO is complicated by various factors. Poor oral hygiene, associated plaque accumulation and pre-existing gingivitis or periodontitis can cause severe swelling of the gingiva. Local irritants such as poorly fitting dentures or cleaning niches can also lead to inflammation and swelling (Agrawal 2015). Accordingly, these and other factors can lead to gingival overgrowth regardless of the medication taken. Once all external and local irritants have been eliminated and the gingival overgrowth persists, a medication-related cause should be considered. Swollen gingiva can be detected both visually and by measuring the depth of the gingival pockets (Salvi et al. 2023). However, increased pocket depth does not automatically mean swollen gingiva. Periodontitis can cause significantly increased pocket depths, which are not due to excessive tissue swelling but to a loss of periodontal tissue. Swelling, which usually accompanies this condition, is caused by an inflammatory reaction of the tissue and can be completely eliminated by thoroughly cleaning the gingival pockets. If swelling occurs that is not associated with periodontitis, so-called pseudo-pockets may develop, which cannot be explained by a loss of periodontium, but rather by an increase of the amount of tissue (Salvi et al. 2023). These pseudo-pockets cannot be eliminated by thorough cleaning, as they develop independently of inflammation. It can therefore be concluded that a number of factors must be ruled out before a reliable diagnosis of DIGO can be made.

Therefore, the classification scheme for periodontal diseases and conditions developed and published in 2018 should be applied here (Caton et al. 2018). First, a distinction must be made between “Periodontal Health, Gingival Diseases and Conditions”, “Periodontitis” and “Other Conditions Affecting the Periodontium”. Within the first category, gingival diseases must be further separated into “dental biofilm-induced” and “non-dental biofilm-induced”. Formally, according to this classification, “drug-influenced gingival enlargement” belongs to the group of dental biofilm-induced gingival diseases (Murakami et al. 2018). Accordingly, only patients with corresponding biofilm/plaque accumulation and gingival overgrowth can be diagnosed with DIGO. If gingival overgrowth occurs without associated plaque accumulation, other factors from the group of “non-dental biofilm-induced gingival diseases” must be considered. This group includes the following: “genetic/developmental disorders”, “specific infections”, “inflammatory and immune conditions” and “reactive processes”, as well as others (Holmstrup et al. 2018). This classification highlights the fundamental influence of dental biofilm on the development and severity of DIGO.

Most of the drugs analysed that lead to gingival overgrowth belong to the group of calcium channel blockers, followed by anticonvulsants, immunosuppressants and other drug classes (Fig. S7). It is also worth noting that the lead of calcium channel blockers compared to the other classes is enormous. The difference of almost 45% between calcium channel blockers and anticonvulsants shows how many patients are treated with these drugs and develop gingival overgrowth. Nevertheless, the results must be seen in the context of the DDDs of the individual drug classes and the prevalence with which this adverse effect occurs. Among the calcium channel blockers, amlodipine was the most frequently taken drug, followed by nifedipine (Fig. 2). This is hardly surprising when one considers the high prescription figures for amlodipine from the current AVR. Immunosuppressants and anticonvulsants are far behind with a difference of 14% to just under 25%. In the experience of the respondents, amlodipine is also the drug most frequently associated with gingival overgrowth (Fig. S8).

However, these results are in contrast to model calculations based on information from the package leaflets of various active ingredients. For example, Ried and Seifert were able to show that valproate in particular should lead to significantly more gingival overgrowth if the current prescriptions and the prevalence stated in the package leaflet are taken as a basis. The question now arises as to how these absolutely contradictory results can come up. It is possible that valproate has not yet moved into the focus of many practitioners, so that gingival overgrowth is rarely associated with this active ingredient.

In addition to valproate, there is reason to suspect that other active substances that have been shown to trigger DIGO are unknown to many dentists and physicians in connection with this ADR and therefore cannot be linked to it. Furthermore, it is possible that in some patients, the symptoms are so mild that they are not clinically noticeable or do not cause any problems for the patient and therefore do not necessarily require treatment. Both possibilities can lead to significant under-reporting of the ADR. In addition, it is often difficult to clinically distinguish between DIGO and gingival overgrowth that is not associated with certain drugs. Since external factors such as the patient’s oral hygiene or existing periodontitis contribute significantly to the severity of gingival overgrowth, it is conceivable that the same active ingredient may lead to DIGO in one patient but not in another. These interindividual factors make it difficult to determine the exact prevalence of DIGO.

In the case of polypharmacy in patients, another active substance could therefore be seen as the cause, although it may be due to valproate. Another explanation could be that the prevalence rates stated in the package inserts of the other active substances investigated are too low and that gingival overgrowth occurs much more frequently in reality than expected. In order to clarify this question conclusively, further studies are required that directly compare the individual active ingredients under controlled conditions.

Several factors could be considered to justify the results of the undertaken survey. Significantly higher prescription rates for amlodipine could lead to a higher prevalence of amlodipine-induced gingival overgrowth compared to the other drugs considered. The largest population group that regularly visits the dentist tends to be older, pre-diseased patients whose pre-existing conditions require the use of calcium channel blockers. This could lead to the overrepresentation of amlodipine in this study. On the other hand, the prescription rates of nifedipine are several times lower than those of amlodipine, but the reported percentage is not even half of that of amlodipine. This suggests that the prevalence of nifedipine-induced gingival overgrowth must be higher than the prevalence of amlodipine-induced overgrowth.

Taking the results from Fig. 3 into account, it becomes clear that it is probably not only the specific medication that plays a major role in whether or not patients develop drug-induced gingival overgrowth. It also depends on general oral hygiene, polypharmacy with possibly multiple medications that increase the risk of developing overgrowth and other unknown factors that increase individual susceptibility. It is therefore important to inform affected patients about their individual options for improving their condition and to guide them towards the best possible oral hygiene. In addition, it can be deduced that there are certain groups of patients who have a higher risk of developing gingival overgrowth than others (Fig. S3).

With regard to the age of the affected patients, the following distribution can be determined. The largest group is 65 years and older or between 40 and 59 years old. This is followed by the second largest group, which is between 60 and 64 years old. The proportion of patients who are under 39 or even under 25 is significantly lower. The smallest group of patients is between 6 and 17 years old (Fig. S10). To summarise, the risk of developing gingival overgrowth increases with age. This is presumably due to the increased likelihood of taking one or more of the triggering medications as well as a decline in oral hygiene.

In addition to age, the patient’s gender could also be a relevant factor in the development of this adverse drug reaction. However, the results of the survey show that the underlying gender has no significant influence on the development of drug-induced gingival overgrowth (Fig. S4). In summary, it can be concluded that certain risk factors such as age, polypharmacy, oral hygiene and individual susceptibility lead to an increased tendency to develop DIGO which is also consistent with studies such as Seymour et al. (2000).

Probably one of the most important questions for patients and treating dentists alike is whether the treatment of the proliferation is successful or not. In order to carry out successful treatment, it is important to identify and eliminate the cause of the symptoms. In most cases, the interviewees succeeded in doing this (Fig. S5). One possible treatment option is to discontinue or change the medication that is the suspected trigger. It was shown that the treatment was successful in the majority of the cases examined and that a change of medication also led to a complete resolution of the gingival overgrowth in many cases (Fig. S6,4). This result proves that certain medications are undoubtedly the cause of this symptom and that causal therapy by discontinuing the causative drug should be considered whenever possible. If the patient’s general condition does not permit such a measure, surgical treatment options and intensive improvement of oral hygiene should be prioritised (Fig. S6).

If changing the triggering medication or surgically removing the excessive tissue is not possible or too risky, the following treatment protocol can achieve a significant improvement in the patient’s situation. At the beginning of the treatment, the patient should be given detailed information about their situation and precise instructions on how to establish optimal oral hygiene. This should be followed by thorough supragingival cleaning, removing all plaque and tartar from the teeth. After that, all subgingival plaque and calculus should be removed (“scaling and root planing”). Systemic antibacterial drugs such as clindamycin can be used to support the treatment (Lv et al. 2025). According to the current S3 guideline, in cases of additional periodontitis, follow-up treatment may be necessary every 3–6 months, depending on the stage. This involves continuous supragingival cleaning and the measurement of periodontal parameters such as pocket depth, gingival recession, degree of tooth loosening and furcation involvement (Sanz et al. 2020). The aim of the therapy is to create a non-inflammatory oral environment and thereby reduce gingival overgrowth.

However, it should be noted that there is no specific guideline for DIGO, which is why practitioners must rely on the general periodontitis guideline in order to adequately treat their patients.

Survey results show that the dentists interviewed acted in accordance with the treatment recommendations described above (Fig. S6). The responses received, in descending order, were as follows: “oral hygiene instructions”, “medication change”, “surgical”, “wait and see” and “other”. This indicates a sequence of treatment from non-invasive to invasive based on the three most frequently registered responses. Although the survey did not explicitly ask about this, it can be assumed that, in addition to verbal instructions, supra- and subgingival cleaning was also performed on patients to eliminate oral inflammation. The current guidelines and recommendations from the literature are consistent with this approach, as optimising oral hygiene can significantly reduce DIGO in many cases. Overall, it appears that the majority of respondents are well informed about recommended treatment protocols and also implement them in practice.

Looking at the mechanisms of action of the drugs that may lead to the triggering of gingival overgrowth, the majority of respondents were not aware of any underlying mechanism (Fig. S12). If the responses to the question about the mechanism of action are reduced by the answer ‘unknown’, the following distribution emerges: a ‘combination of the mechanisms mentioned’ was chosen most frequently, followed by ‘inflammation-induced upregulation of cytokines’. A ‘plaque-associated inflammation of the soft tissue’ was then suspected, followed by ‘impaired connective tissue breakdown’ and ‘triggering of inflammatory processes by toxic drug levels in the sulcus fluid’. Even less frequently, only ‘other mechanism’ and ‘cation current reduced by the drug’ were selected. This distribution ultimately emphasises the prevailing uncertainty regarding the mechanism of development of drug-induced gingival overgrowth. The survey results described above indicate that there could be a substantial knowledge gap among respondents regarding the molecular mechanisms underlying the development of DIGO (Fig. S12). Continuous professional development and reading current literature appear to be essential in order to remain up to date with the latest insights and to be able to treat diseases and symptoms adequately.

For a further exploratory analysis, various survey results were compared with one another. Although the survey did not specify which particular drug class patients in the different age groups were taking, it is possible to make some assumptions based on the results. When comparing the survey results, starting with the most frequently selected answers in descending order, the following stands out: Most patients with DIGO were 65 years of age or older, and the most commonly prescribed drug class was calcium channel blockers. Although no direct correlation between the responses was identified in the survey, it is reasonable to suspect that the number of patients treated with calcium antagonists increases dramatically with age. The second most frequently affected patients were between 40 and 59 years old, and a combination of several drug classes was identified. Patients aged 18–25 were the least frequently affected, and drugs that did not belong to the classes of calcium antagonists, anticonvulsants or immunosuppressants were identified as triggers of DIGO (Fig. S13). From this, it can be concluded that with increasing age, both the number of patients on polypharmacy and the number of patients treated with calcium antagonists likely rise dramatically.

Following this analysis, the respective drug classes and the emerging symptom profile were compared. In descending order, patients most commonly took calcium channel blockers, combinations of several drug classes, anticonvulsants and immunosuppressants (Fig. S14). The most frequently observed symptoms include the following: swollen gingiva, bleeding gingiva, difficulty with oral hygiene, compromised aesthetics and painful gingiva. Bad breath, intraoral burning and eating difficulties may also occur, though much less frequently. This distribution indicates which symptoms are most likely to occur when a patient is treated with the aforementioned drugs. Reversely, a detailed medical history should be taken into account when patients present with these symptoms without a clear cause.

When comparing treatment outcomes with the treatment modality for DIGO, the following conclusions can be drawn. Most of the dentists surveyed were successful with their treatment, and the most frequently chosen treatment option was oral hygiene instruction. The second-largest group of respondents did not treat the DIGO themselves, followed by those who did not know whether their treatment was successful or not. The smallest group was unsuccessful with their treatment. After oral hygiene instruction, the second most common approach was a medication change, followed by surgical treatment of DIGO. The least common approaches were to wait and see or to choose another treatment option. From this distribution, it can be assumed that oral hygiene instruction alone has a high probability of leading to treatment success, which emphasises the importance of dental biofilm in DIGO. A change in medication and surgical treatment also appears to have a high success rate, although these therapeutic measures can also be carried out through interdisciplinary collaboration, which may explain the high proportion of dentists who did not treat the present DIGO cases themselves (Fig. S15).

Current research also shows that although there is a common mechanism in the blockade of ion channels, the exact relationship between individual active substances and the development of DIGO has not been conclusively clarified (Droździk and Droździk 2023). While there is a significant correlation between plaque accumulation, plaque-induced gingival inflammation and DIGO, this does not mean that DIGO does not also occur in plaque- or inflammation-free oral conditions. On the contrary, there appears to be a significant genetic influence that causes particularly susceptible fibroblasts in certain patients, while other patients appear to be considerably more resistant to gingival overgrowth-inducing drugs. Depending on the active ingredient, different histological changes in the tissue can be observed. While phenytoin primarily causes fibrotic changes, CCBs such as nifedipine can cause a mixture of inflammatory and fibrotic changes, and immunosuppressants such as ciclosporin lead to predominantly inflammatory changes (Droździk and Droździk 2023). In addition to these differences, it is also unclear why different drugs in the same drug class lead to DIGO with varying degrees of severity and frequency. For example, DIGO occurs significantly more frequently in patients taking amlodipine and nifedipine than in those taking felodipine (Vidal et al. 2018). Therefore, various unanswered questions remain that should be further investigated in future research.

## Limitations

The data collected and analysed corresponds to a sample and may not be comprehensive or complete. The questionnaire was developed by the authors and pre-tested internally but was not formally validated (e.g. cognitive pre-testing, test–retest reliability or expert-panel content validation). In addition, the survey was conducted in a ‘small’ group, which limits the reliability and significance of the results. Furthermore, the very low response rate (~ 0.46%) has to be considered as well. Since only 118 completed questionnaires were available for data analysis, the results presented, in particular possible prevalences and expected DIGO cases in Germany, are only of very limited significance and are, in principle, very difficult to generalise. It must also be taken into account that there may be a significant self-selection bias. This means that mainly dentists who are familiar with the topic of DIGO or who already have considerable prior knowledge in this area completed the survey. This may lead to a significant distortion of the results, as the respondents in particular have observed triggers of DIGO in their patients that they were already aware of and indicated this in the survey. In addition, there were no control mechanisms in place with regard to the selection of participants. Although it can be assumed that the link to the survey distributed by email was only opened by the dentists to whom the email was addressed, the possibility of third-party access (others than dentists) cannot be ruled out. Accordingly, it cannot be verified whether only dentists actually completed the survey conscientiously or if outsiders could have manipulated the results. Furthermore, it must be taken into account that there may be locally varying prescription patterns, which may result in different rates of use of the various active ingredients depending on the region. This means that there may be considerable regional variations in the prevalence of the different active ingredients in inducing gingival overgrowth.

## Conclusions

The survey results show that the majority of respondents (84%) suspect drugs as the cause of gingival overgrowth in their patients. Nevertheless, it must be borne in mind that this likely represents a DIGO-aware subgroup of the German dental community, and the results may therefore be inflated. At the time of onset of GO, amlodipine was the most commonly taken drug (31%), followed by nifedipine (20%) and ciclosporin (7%). Phenobarbital and valproate, on the other hand, were reported particularly rarely, at 1% each. These results are surprisingly low considering that valproic acid is reported to have a prevalence of 30% (Droździk and Droździk 2023) to 81% (PI/SmPC). It must be taken into account that locally varying prescription figures and, in some cases, a lack of knowledge on the part of the respondents may lead to distorted results, and that there may therefore be a high number of unreported cases of patients with valproic acid-induced gingival overgrowth. Among the most important risk factors leading to the development of DIGO are the substance group (50%), oral hygiene (23%) and polypharmacy (10%). In accordance with current treatment recommendations, dentists reported treating their patients with ‘oral hygiene instructions’ (37%), ‘medication changes’ (26%) and ‘surgery’ (23%). As a result, roughly 70% of respondents were able to successfully treat their patients’ DIGO. In particular, a change of medication led to a significant improvement or complete reduction of gingival overgrowth in 41% of cases. The drug classes most frequently selected as the cause by respondents were as follows: calcium channel blockers (54%), anticonvulsants (9%) and immunosuppressants (8%). In conclusion, the majority of respondents (57%) were unaware of the exact mechanism behind DIGO. And although not all aspects have been conclusively clarified at the molecular level, there is a common mode of action by which the various active substances lead to gingival overgrowth.

## Further perspectives

For future research, there is a growing list of drugs that are suspected of causing gingival diseases, including gingival overgrowth. However, there is still no detailed review or confirmation of this link. In addition, the exact mechanisms of drug-induced gingival overgrowth are still not conclusively understood, so further studies are needed to understand these complex relationships.

## Supplementary Information

Below is the link to the electronic supplementary material.

ESM1 (PDF 215 KB)

ESM2 (DOCX 20.0 KB)

ESM3 (DOCX 2.06 MB)

## Data Availability

All source data for this study are available upon reasonable request from the authors.

## Abbreviations

ADR: Adverse drug reaction
AVR: Arzneiverordnungs-Report (Drug Prescription Report)
DDD: Defined daily dose
DIGO: Drug-induced gingival overgrowth
SmPC: Summary of product characteristics
