# Supplementary material for: Drug-induced gingival overgrowth: evaluation of an online survey
Source: Naunyn Schmiedebergs Arch Pharmacol. 2026 Jun 20;399(12):19187–99. doi: 10.1007/s00210-026-05600-6 (PMC13593694; doi:10.1007/s00210-026-05600-6)
Supplement: Supplementary file 1 [file 210_2026_5600_MOESM1_ESM.pdf]

Seite 01

Herzlich Willkommen bei dem Fragebogen zum Thema Gingivahyperplasie!

Bitte beantworten Sie die nachfolgenden Fragen nach bestem Wissen und Gewissen. Es werden hierbei keine personenbezogenen Daten erhoben und Ihre Antworten können Ihrer Person nicht zugeordnet werden.

Mit einem Klick auf "Weiter" stimmen Sie der Teilnahme zu.

Vielen Dank!

PA01

1. Wie häufig haben sich bereits Patienten mit einer Gingivahyperplasie bei Ihnen vorgestellt?

- ◯ nie
- ◯ selten
- ◯ gelegentlich
- ◯ häufig
- ◯ sehr oft
- ◯ keine Angabe

PA02

2. Gibt es spezielle Patientengruppen, bei denen Sie ein vermehrtes Auftreten einer Gingivahyperplasie beobachten?

- ◯ ja
- ◯ nein
- ◯ unbekannt

PA07

3. Sind Ihrer Erfahrung nach häufiger Frauen oder Männer von der Gingivahyperplasie betroffen?

- ◯ Männer sind häufiger betroffen
- ◯ Frauen sind häufiger betroffen
- ◯ Männer und Frauen sind ungefähr gleich häufig betroffen
- ◯ unbekannt

PA03

4. Zu welcher Altersgruppe gehören Ihre Patienten mit Gingivahyperplasie:

- ◯ unter 1 Jahr
- ◯ 1-5 Jahre
- ◯ 6-17 Jahre
- ◯ 18-25 Jahre
- ◯ 26-39 Jahre
- ◯ 40-59 Jahre
- ◯ 60-64 Jahre
- ◯ 65 Jahre und älter
- ◯ unbekannt

RF01

5. Gibt es Ihrer Einschätzung nach bestimmte Risikofaktoren, die die Entstehung von arzneimittelassoziierter Gingivahyperplasie begünstigen könnten?

- ◯ ja
- ◯ nein
- ◯ unbekannt

RF02

6. Welcher der folgenden Faktoren ist Ihrer Meinung nach der wichtigste Risikofaktor für die Entstehung einer Gingivahyperplasie?

- ◯ die Medikamentengruppe (Calciumkanalblocker, Immunsuppressiva, Antikonvulsiva)
- ◯ die Mundhygiene
- ◯ das Alter
- ◯ das Geschlecht
- ◯ die individuelle Empfänglichkeit für Gingivahyperplasien
- ◯ Polypharmazie
- ◯ andere
- ◯ unbekannt

RF03

7. Welcher der folgenden Faktoren ist Ihrer Meinung nach der zweitwichtigste Risikofaktor für die Entstehung einer Gingivahyperplasie?

- ○ die Medikamentengruppe (Calciumkanalblocker, Immunsuppressiva, Antikonvulsiva)
- ○ die Mundhygiene
- ○ das Alter
- ○ das Geschlecht
- ○ die individuelle Empfänglichkeit für Gingivahyperplasien
- ○ Polypharmazie
- ○ andere
- ○ unbekannt

8. Haben Sie die Gingivahyperplasie bei Ihren Patienten therapiert? TH01

- ○ ja
- ○ nein

TH06

9. Welche Behandlungsoptionen setzen Sie bei Ihren Patienten mit Gingivahyperplasie ein? (Mehrfachauswahl möglich)

- ☐ medikamentöse Umstellung
- ☐ Mundhygieneinstruktionen
- ☐ chirurgisch
- ☐ abwartend
- ☐ andere

TH02

10. War Ihre Therapie erfolgreich?

- ◯ ja
- ◯ nein
- ◯ unbekannt
- ◯ ich habe die Gingivahyperplasie nicht selbst therapiert

TH04

11. Bestand Ihre Therapie aus einer medikamentösen Umstellung der Patienten?

- ◯ ja
- ◯ nein

TH05

12. Ist die Gingivahyperplasie unter absetzen/ wechseln des Medikamentes abgeklungen bzw. hat sich vollständig zurückgebildet?

- ◯ ja
- ◯ nein
- ◯ unbekannt

PA04

13. Ist Ihren Patienten die Gingivahyperplasie selbst aufgefallen?

- ○ ja
- ○ nein
- ○ unbekannt
- ○ bei meinen Patienten lag keine Gingivahyperplasie vor

PA06

14. Welche Symptome treten Ihrer Erfahrung nach häufig bei Patienten mit arzneimittelassoziierter Gingivahyperplasie auf? (Mehrfachauswahl möglich)

- ☐ blutendes Zahnfleisch
- ☐ geschwollenes Zahnfleisch
- ☐ Mundgeruch
- ☐ erschwerte Mundhygiene
- ☐ erschwerte Aussprache
- ☐ erschwerte Nahrungsaufnahme
- ☐ eingeschränkte Ästhetik
- ☐ Missempfindungen
- ☐ schmerzhaftes Zahnfleisch
- ☐ intraorales „brennen“ (Burning Mouth Syndrome)
- ☐ andere Symptome
- ☐ keines der genannten Symptome

PA05

15. Gab es einen Behandlungswunsch bzgl. der Gingivahyperplasie von Seiten Ihrer Patienten?

- ○ ja
- ○ nein
- ○ unbekannt

AU01

16. Konnten Sie den Auslöser der Gingivahyperplasie feststellen?

- ja
- nein
- unbekannt

AR01

17. Haben Sie den Verdacht, dass die Gingivahyperplasie potenziell arzneistoff-induziert/ assoziiert sein könnte?

- ja
- nein
- unbekannt

AR02

18. Gibt es Ihrer Meinung nach bestimmte Medikamente, die häufiger zu einer Gingivahyperplasie führen als Andere?

- ja
- nein
- unbekannt

AR04

19. Wenn ja zu welcher Wirkstoffklasse gehört der auslösende Arzneistoff, der bei Ihren Patienten zum Auftreten einer Gingivahyperplasie geführt hat?

- ○ Calciumantagonist/ Calciumkanalblocker (L-Typ) (z.B.: Amlodipin, Felodipin, Nifedipin, Lercanidipin etc.)
- ○ Antikonvulsiva / Antiepileptika / Anfallssuppressiva (z.B.: Carbamazepin, Phenytoin, Valproat, Phenobarbital etc.)
- ○ Immunsuppressiva (z.B: Cyclosporin A)
- ○ andere Wirkstoffklasse
- ○ Kombination mehrerer Wirkstoffklassen
- ○ unbekannt

AR10

20. Welcher Arzneistoff im speziellen wurde von den Patienten zum Zeitpunkt des Auftretens der Gingivahyperplasie eingenommen? (Mehrfachauswahl möglich)

- ☐ Amlodipin
- ☐ Nifedipin
- ☐ Lercanidipin
- ☐ Felodipin
- ☐ Phenytoin
- ☐ Valproat
- ☐ Carbamazepin
- ☐ Phenobarbital
- ☐ Cyclosporin A
- ☐ unbekannt

AR11

21. Welcher Arzneistoff löst Ihrer Erfahrung nach am häufigsten Gingivahyperplasien aus?

- ◯ Amlodipin
- ◯ Nifedipin
- ◯ Lercanidipin
- ◯ Felodipin
- ◯ Phenytoin
- ◯ Valproat
- ◯ Carbamazepin
- ◯ Phenobarbital
- ◯ Cyclosporin A
- ◯ unbekannt

22. Welcher Arzneistoff löst Ihrer Erfahrung nach am zweithäufigsten Gingivahyperplasien aus? AR12

- ◯ Amlodipin
- ◯ Nifedipin
- ◯ Lercanidipin
- ◯ Felodipin
- ◯ Phenytoin
- ◯ Valproat
- ◯ Carbamazepin
- ◯ Phenobarbital
- ◯ Cyclosporin A
- ◯ unbekannt

WI01

23. Sind Ihnen Wirkmechanismen von Arzneistoffen bekannt die möglicherweise zur Auslösung einer Gingivahyperplasie führen?

- ○ ja
- ○ nein

WI02

24. Welchen der nachfolgend genannten Mechanismen würden Sie als am Wahrscheinlichsten für die Entstehung einer Gingivahyperplasie auswählen?

- ○ fehlerhafte Kollagenase-Aktivität auf Grund von mangelhafter Folsäureaufnahme
- ○ ein durch das Medikament verringerter Kationenstrom
- ○ ein gestörter Bindegewebsabbau
- ○ Erhöhung des Keratinozytenwachstumsfaktors (KGF)
- ○ Auslösung entzündlicher Prozesse durch toxische Arzneistoffspiegel in der Sulkusflüssigkeit
- ○ entzündlich bedingte Hochregulation von diversen Zytokinen wie z.B.: TGF-β1 (Transforming Growth Factor β)
- ○ eine plaqueassoziierte Entzündung des Weichgewebes
- ○ Kombination der genannten Mechanismen
- ○ keiner der genannten Mechanismen
- ○ anderer Mechanismus
- ○ unbekannt

Letzte Seite

Vielen Dank für Ihre Teilnahme! Wir möchten uns ganz herzlich für Ihre Zeit und Mithilfe bedanken. Ihre Antworten wurden gespeichert, Sie können das Browser-Fenster nun schließen.
