# Supplementary material for: Drug-induced gingival overgrowth: evaluation of an online survey
Source: Naunyn Schmiedebergs Arch Pharmacol. 2026 Jun 20;399(12):19187–99. doi: 10.1007/s00210-026-05600-6 (PMC13593694; doi:10.1007/s00210-026-05600-6)
Supplement: Supplementary file 2 [file 210_2026_5600_MOESM2_ESM.docx]

Welcome to the questionnaire on gingival hyperplasia!

Please answer the following questions to the best of your knowledge and belief.

No personal data will be collected and your answers cannot be traced back to you personally.

By clicking on “Continue,” you agree to participate.

Thank you very much!

1. How often have patients with gingival hyperplasia approached you?

never

rarely

occasionally

frequently

very often

no information provided

2. Are there specific patient groups in which you observe an increased incidence of gingival hyperplasia?

yes

no

unknown

3. In your experience, are women or men more frequently affected by gingival hyperplasia?

Men are more frequently affected

Women are more frequently affected

Men and women are affected with approximately equal frequency

Unknown

4. What age group do your patients with gingival hyperplasia belong to?

Under 1 year

1-5 years

6-17 years

18-25 years

26-39 years

40-59 years

60-64 years

65 years and older

Unknown

5. In your opinion, are there certain risk factors that could promote the development of drug-associated gingival hyperplasia?

yes

no

unknown

6. In your opinion, which of the following is the most important risk factor for the development of gingival hyperplasia?

the group of medications (calcium channel blockers, immunosuppressants,

anticonvulsants)

oral hygiene

age

gender

individual susceptibility to gingival hyperplasia

polypharmacy

other

unknown

7. Which of the following factors do you consider to be the second most important risk factor for the development of gingival hyperplasia?

the group of medications (calcium channel blockers, immunosuppressants,

anticonvulsants)

oral hygiene

age

gender

individual susceptibility to gingival hyperplasia

polypharmacy

other

unknown

8. Have you treated the gingival hyperplasia of your patients?

Yes

No

9. What treatment options do you use for your patients with gingival hyperplasia? (Multiple selections possible)

change of medication

oral hygiene instructions

surgical

wait-and-see approach

other

10. Was your therapy successful?

Yes

No

Unknown

I did not treat the gingival hyperplasia myself.

11. Did your therapy consist of changing the patients' medication?

Yes

No

12. Has the gingival hyperplasia subsided or completely disappeared after discontinuing/changing the medication?

Yes

No

Unknown

13. Have your patients noticed the gingival hyperplasia themselves?

Yes

No

Unknown

My patients did not have gingival hyperplasia.

14. In your experience, what symptoms frequently occur in patients with drug-induced gingival hyperplasia? (Multiple selections possible)

Bleeding gingiva

Swollen gingiva

Bad breath

Difficult oral hygiene

Difficult pronunciation

Difficult eating

Limited aesthetics

Discomfort

Painful gingiva

Intraoral “burning” (burning mouth syndrome)

Other symptoms

None of the above symptoms

15. Did your patients request treatment for gingival hyperplasia?

Yes

No

Unknown

16. Were you able to determine the cause of the gingival hyperplasia?

Yes

No

Unknown

17. Do you suspect that the gingival hyperplasia could potentially be drug-induced/associated?

Yes

No

Unknown

18. In your opinion, are there certain medications that lead to gingival hyperplasia more often than others?

Yes

No

Unknown

19. If so, to which class of active substances does the triggering drug belong that led to the occurrence of gingival hyperplasia in your patients?

Calcium antagonists/calcium channel blockers (L-type) (e.g., amlodipine, felodipine,

nifedipine, lercanidipine, etc.)

Anticonvulsants/antiepileptics/seizure suppressants (e.g., carbamazepine,

phenytoin, valproate, phenobarbital, etc.)

Immunosuppressants (e.g., cyclosporine A)

Other classes of drugs

Combination of several classes of drugs

Unknown

20. Which specific drug was being taken by patients at the time of onset of gingival hyperplasia? (Multiple selections possible)

amlodipine

nifedipine

lercanidipine

felodipine

phenytoin

valproate

carbamazepine

phenobarbital

cyclosporine

Unknown

21. In your experience, which drug most commonly causes gingival hyperplasia?

amlodipine

nifedipine

lercanidipine

felodipine

phenytoin

valproate

carbamazepine

phenobarbital

cyclosporine

Unknown

22. In your experience, which drug is the second most common cause of gingival hyperplasia?

amlodipine

nifedipine

lercanidipine

felodipine

phenytoin

valproate

carbamazepine

phenobarbital

cyclosporine

Unknown

23. Are you aware of any mechanisms of action of drugs that may trigger gingival hyperplasia?

Yes

No

24. Which of the following mechanisms would you select as the most likely cause of gingival hyperplasia?

Impaired collagenase activity due to insufficient folic acid intake

A reduction in cation flow caused by the medication

Impaired connective tissue breakdown

Increase in keratinocyte growth factor (KGF)

Triggering of inflammatory processes by toxic drug levels in the sulcus fluid

Inflammation-induced upregulation of various cytokines such as TGF-β1 (transforming growth factor β)

Plaque-associated inflammation of the soft tissue

Combination of the above mechanisms

None of the above mechanisms

Other mechanism

Unknown

Thank you for participating! We would like to express our sincere gratitude for your time and assistance. Your answers have been saved; you can now close the browser window.
