# Supplementary material for: Drug-induced gingival overgrowth: evaluation of an online survey
Source: Naunyn Schmiedebergs Arch Pharmacol. 2026 Jun 20;399(12):19187–99. doi: 10.1007/s00210-026-05600-6 (PMC13593694; doi:10.1007/s00210-026-05600-6)
Supplement: Supplementary file 3 [file 210_2026_5600_MOESM3_ESM.docx]

**Supplemental material:**

**Drug-induced gingival overgrowth:**

**Evaluation of an online survey**

**Philipp Friedrich Georg Ried**

**and**

**Roland Seifert**

**Institute of Pharmacology
Hannover Medical School
D-30625 Hannover, Germany
Corresponding author: Roland Seifert
seifert.roland@mh-hannover.de**

**“Do you suspect that the gingival hyperplasia could potentially be drug-induced/associated?”**

 ***Fig. S1*** Presentation of the registered responses to the question of whether the gingival overgrowth could potentially be drug-induced. Evaluation of the possible answers in absolute numbers and as a percentage.

**“Were you able to determine the cause of the gingival hyperplasia?”**

 ***Fig. S2*** Evaluation of whether the trigger of the gingival overgrowth could be determined. Display in per cent and absolute numbers

**“Are there specific patient groups in which you observe an increased incidence of gingival hyperplasia?”**

***Fig. S3*** Presentation of the survey results on the question of whether specific patient groups are more frequently affected by gingival overgrowth. Overview of the registered answers in per cent and absolute numbers

**“In your experience, are women or men more frequently affected by gingival overgrowth?”**

 ***Fig. S4*** Presentation of the answers given to the question of whether men or women suffer more frequently from gingival overgrowth. Overview in per cent and absolute numbers

**“Was your therapy successful?”**

***Fig. S5*** Presentation of the answers to the question of whether the therapy was successful or not. In per cent and absolute numbers

**"Which treatment options do you use for your patients with gingival hyperplasia? (multiple choice possible)"**

***Fig. S6*** Overview of the treatment options carried out by the respondents. It was possible to select multiple answers to this question. Presentation of the answers given in absolute numbers and in percent

**“Which drug class does the triggering drug that led to the occurrence of gingival hyperplasia in your patients belong to?”**

***Fig. S7*** Evaluation of the indicated active substance classes that led to gingival overgrowth. Presentation as a percentage and in absolute numbers

**“In your experience, which drug most frequently triggers gingival hyperplasia?”**

***Fig. S8*** Presentation of which drug most frequently leads to gingival overgrowth according to experience. In per cent and absolute numbers

**“How often have patients with gingival hyperplasia already consulted you?”**

***Fig. S9*** Overview of the frequency with which patients suffering from gingival overgrowth introduced themselves. Presentation of the possible answers in per cent and absolute numbers

**“Which age group do your patients with gingival hyperplasia belong to?”**

***Fig. S10*** Visualisation of the age groups of patients presenting with gingival overgrowth. Overview of registered responses in absolute numbers and in per cent

**“In your experience, which symptoms frequently occur in patients with drug-induced gingival hyperplasia? (multiple choice possible)“**

***Fig. S11*** Overview of the symptoms described in patients with gingival overgrowth. Presentation of the registered answers in per cent and absolute numbers. It was possible to select several symptoms at the same time for this question.

**“Which of the following mechanisms would you select as the most likely for the development of gingival hyperplasia?”**

***Fig. S12*** Overview of the mechanisms selected by the participants that are most likely to lead to the development of gingival overgrowth. Presentation of the answers given in per cent and absolute numbers

***Fig. S13*** Comparison of survey results regarding the age of patients with DIGO and the most commonly taken medication group. The results are sorted from highest to lowest. There is no correlation between age group and medication group; the data presented is for illustrative purposes only.

 ***Fig. S14*** Visualization of survey results regarding the symptom profile of patients with DIGO and the most commonly taken medication group. The results are sorted from highest to lowest. The data presented is for illustrative purposes only.

 ***Fig. S15*** Comparison of survey results regarding the treatment outcome of patients with DIGO and the treatment modality. The results are sorted from highest to lowest. The data presented is for illustrative purposes only.

| manufacturer | code |
| --- | --- |
| Hexal AG | 1 |
| Teva Pharmaceutical Industries Limited | 2 |
| Ratiopharm GmbH | 3 |

***Table S1*** Naming the pharmaceutical manufacturers with the codes used in the paper

| Section | Moride et al., 1997 | Alvarez-Requejo et al., 1998 | Ried & Seifert, this study |
| --- | --- | --- | --- |
| Aim | Assessing the quantitative level of under-reporting of ADRs in general practice. | Quantify the extent of under-reporting and assess influencing factors. | Assessing which drugs potentially lead to gingival overgrowth and what prevalence can be expected. |
| Methods | 81 general practitioners in the region of the Bordeaux pharmacovigilance centre were surveyed to obtain all observed ADRs on 1 work day per month for three consecutive months. All observed ADRs were compared to the ADRs reported to the Bordeaux pharmacovigilance centre in the same time period. | ADRs collected from 106 general practitioners through a survey were compared with spontaneous reports received by the regional pharmacovigilance centre during a 12-month reference period. | During 2 and a half months dentists in 5 federal states of Germany were asked to complete a survey about their experience with drug induced gingival overgrowth. Focussing on the specific drugs taken by their patients as well as numerous influencing factors. The data collected from 118 questionnaires was compared with information provided by scientific literature, package inserts and SmPCs. |
| Results | Only 1 out of every 24433 ADRs per year is believed to be reported to the pharmacovigilance centre by general practitioners. The observed Under- reporting was lowest for serious, unlabelled effects and drugs recently marketed. | The measured under-reporting rate was 1144. The under-reporting was lower for recently marketed drugs and unlabelled effects as well as severe effects. | It is very difficult to determine an exact prevalence for the occurrence of DIGO for certain active substances. The compared data showed a broad, very heterogeneous result. Overall, the adverse drug reaction under consideration is a very relevant topic for German dentists, which is highly likely to be observed in everyday working life. |
| Differences | All ADRs were considered no matter its seriousness and labelling. | All effects that were presumably linked to drugs taken by the patient were considered, no matter the severity or novelty. | Only drug-induced gingival overgrowth was considered in the selected time period. The data were not compared with figures from pharmacovigilance centres. |

***Table S2*** Comparison of various papers that investigated adverse drug reactions by means of surveys and attempted to show their frequency.
